# Effect of Organic Cage Nucleating Agent Structure on Nucleating Efficiency and the Structure-Property Relationship

**DOI:** 10.3390/polym12091975

**Published:** 2020-08-31

**Authors:** Yuhui Zhou, Li He, Wei Gong

**Affiliations:** 1 School of Chemistry and Chemical Industry of Guizhou University, Guiyang 550025, China; huihuisabrina@aliyun.com; 2 Department of Polymer Material and Engineering, College of Materials and Metallurgy, Guizhou University, Guiyang 550025, China; 3 National Engineering Research Center for Compounding and Modification of Polymer Materials, Guiyang 550025, China; 4 School of Materials and Architectural Engineering of Guizhou Normal University, Guiyang 550025, China

**Keywords:** heterogeneous nucleation, cell morphology, injection molding foaming, composite materials, visualization

## Abstract

Three types of organic cage compounds, namely, cucurbit[6]uril (Q[6]), hemicucurbit[6]uril (HQ[6]), and *β*-cyclodextrin (BC), with different cavity structures as heterogeneous nucleation agents were selected for a polypropylene (PP) foaming injection molding process. The experimental results showed that Q[6] with a “natural” cavity structure possessed the best nucleation efficiency of these three cage compounds. The nucleation mechanism of organic cage compounds was explored through classical nucleation theory, molecular structure, and in situ visual injection molding analysis.

## 1. Introduction

The specific strength [1,2,3,4,5] and dimensional stability of foaming materials [6] are directly related to cell density, which is determined by the number of cells present during the foaming process. Therefore, the quality of foaming materials can be effectively improved by regulating nucleation in the foaming process [7,8,9,10,11,12].

According to classical theory [13,14,15,16], the nucleation process of gas in melt proceeds in two modes, namely, homogeneous nucleation and heterogeneous nucleation. In homogeneous nucleation, the second-phase component in a single homogeneous phase forms a stable second phase through accumulation and dispersion fluctuation over a critical size. Homogeneous nucleation occurs when no nucleating agent is contained in the polymer melt, or when the content of the nucleating agent is lower than its solubility in the melt. However, in actual material preparation, all types of additives are used, impurities are common (except for gas and polymer), and various internal tissue defects emerge. These external particles or rough surfaces contribute to heterogeneous nucleation in the foaming process. With regard to the phase change process during material processing and preparation, almost all nucleation behaviors are non-uniform nucleation processes. Thus, studying heterogeneous nucleation is crucial for the preparation of plastic foaming materials.

Research has shown that adding nano-inorganic particles to a polymer as a heterogeneous nucleating agent can effectively improve the quality of the cell morphology, as well as the impact toughness and heat insulation performance of microfoaming materials [17,18,19,20]. However, the nucleation efficiency of nano-inorganic powders is reduced because of their high surface density and easy agglomeration [21,22]. In addition, the compatibility between an inorganic nucleating agent and a matrix resin is poor, and excessive addition degrades the performance of the foaming materials. Therefore, the design and development of highly effective organic nucleating agents with a special structure are essential for improving the foaming behavior and properties of polymers.

Thus far, no study has investigated the application of organic caged compounds as heterogeneous nucleating agents in polymer foaming. In the current study, polypropylene (PP) was used as the polymer substrate. PP and cucurbit[6]uril (Q[6]), hemicucurbit[6]uril (HQ[6]), and *β*-cyclodextrin (BC) composite foaming materials were prepared through microcellular injection. The PP foaming behavior of organic caged and traditional nucleating agents was observed in situ using a visual injection molding device under the condition of adding the same amount of nucleating agent particles. The aim was to analyze and compare the nucleating performance of each organic cage nucleating agent. The formation and growth of cells during the foaming process of each composite material were summarized, and the heterogeneous nucleation mechanism of the caged compound was verified.

## 2. Experimental Setup

### 2.1. Materials

Nano-sized Q[6] and HQ[6] were prepared according to the procedures described in previous literature [23,24]. *β*-cyclodextrin (BC) was of reagent grade and was used without further purification, and silicon dioxide (SiO_2_) was obtained from Sinopharm Group (Shanghai, China). PP T30S with a melt flow index of 3.2 g/10 min at 230 °C/2.16 kg and a density of 0.906 g/cm^3^ was obtained from SINOPEC, Beijing, China. The blowing agent, azodicarbonamide (AC), was obtained from Hanhong Co., Wuhan, China, with a gas production of 220 mL/g.

### 2.2. Nanocomposite Preparation

Q[6], HQ[6], BC, and PP were vacuum dried at 80 °C for 8 h before use. Then, PP with different Q[6], HQ[6], and BC contents was melt-extruded using a twin-screw extruder with an increasing extrusion temperature profile (175~195 °C). The Q[6], HQ[6], and BC contents prepared for the master batch nanocomposites were 0.25, 0.5, 1.0, 2.0, 3.0, 5.0, and 7.0 wt %, and were thereafter coded as PQ0.25, PQ0.5, PQ1.0, PQ2.0, PQ3.0, PQ5.0, and PQ7.0; PH0.25, PH0.5, PH1.0, PH2.0, PH3.0, PH5.0, and PH7.0; and PB0.25, PB0.5, PB1.0, PB2.0, PB3.0, PB5.0, and PB7.0, respectively.

### 2.3. Injection Molding Foaming

The foaming samples were prepared using a microcellular injection foaming molding machine equipped with a volume-adjustable cavity. The extrusion temperature profile from hopper to nozzle was 165~175 °C, and the expansion ratio remained constant, controlled by the sample thickness expanding from 3.5 to 4.0 mm. The gas (N_2_) content was 1%, which was determined by the percentage of AC gas production.

### 2.4. In Situ Foaming Visualization

**Figure 1:**
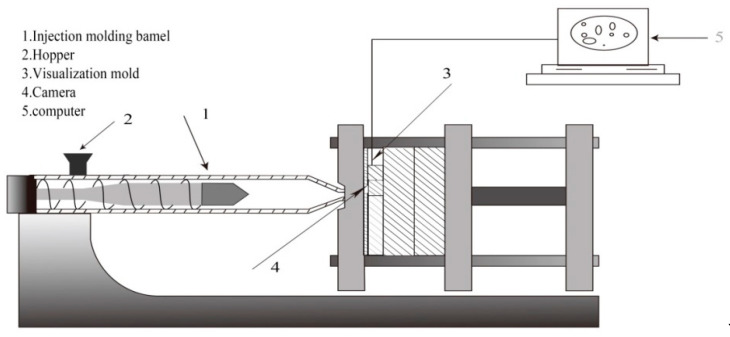
Schematic diagram of the visual injection molding device.

The setup of the batch foaming visualization system, as illustrated in Figure 1, was used to observe the in situ foaming behavior of the aforementioned polymer blowing agent system. The system consisted of a double screw system, a data acquisition system for pressure measurement (i.e., a data acquisition board and a computer), and an optical system (i.e., an objective lens, a light source, and a high-speed camera).

### 2.5. Morphological Analysis

The morphologies of the foamed samples were observed with a scanning electron microscope (SEM) at an accelerating voltage of 25.0 KV (KYKY-2800B, Zhongke Co., Beijing, China). The samples were first frozen in liquid nitrogen for 3 h, and then transversely fractured in a liquid nitrogen atmosphere. Before SEM testing, the samples were sprayed gold for 60 s. Both the cell size and density were determined from the SEM micrographs. The cell size and cell density were calculated via image analysis. The cell density was calculated from the following equation:

(1) $$N={\left[\frac{nM^{2}}{A}\right]}^{\frac{3}{2}}[\frac{\rho }{\rho_{f}}]$$

where *N*_0_ is the cell density (cells/cm^3^), *n* is the number of cells on the SEM micrograph, *M* is the magnification factor, and *A* is the area of the micrograph (cm^2^). The densities of unfoamed (*ρ*) and foamed (*ρ_f_*) samples were measured via the water displacement method, in accordance with ASTM D792.

### 2.6. Transmission Electron Microscope (TEM) Analysis

The morphology of Q[6] was determined by a transmission electron microscope (Tecnai G2 F20, FEI Co., Portland, OR, USA) at 200 KV. Q[6] was prepared using acetone to form a relatively dilute suspension solution, and the solution was then dropped onto the copper grids before the TEM analysis.

## 3. Results and Discussion

### 3.1. Cell Morphology

**Figure 2:**
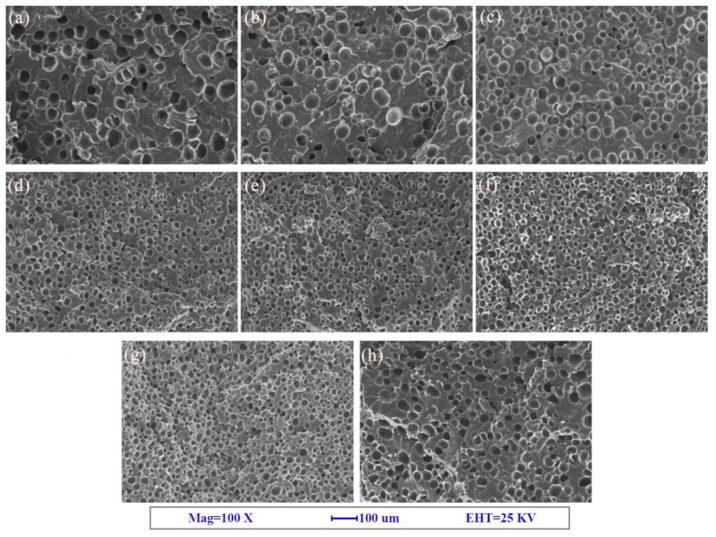
SEM images of the polypropylene (PP) and PP + cucurbit[6]uril (Q[6]) (PQ) composite foams: (**a**) PP, (**b**) PQ0.25, (**c**) PQ0.5, (**d**) PQ1, (**e**) PQ2, (**f**) PQ3, (**g**) PQ5, and (**h**) PQ7.

**Figure 3:**
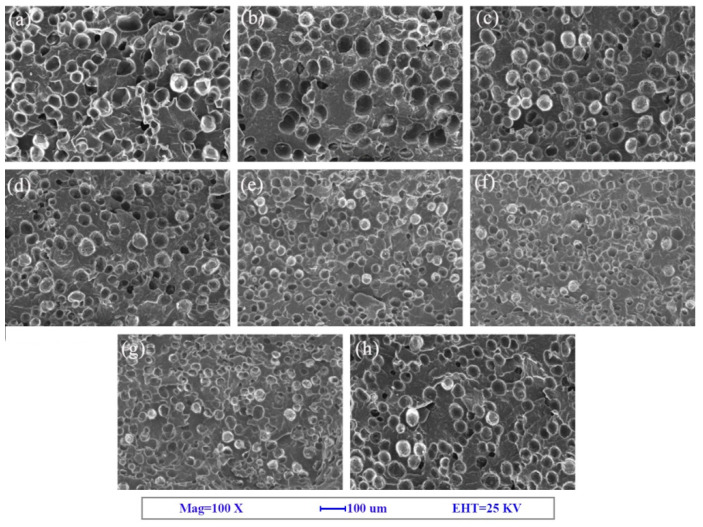
SEM images of the PP and PP + hemicucurbit[6]uril (HQ[6]) (PH) composite foams: (**a**) PP, (**b**) PH0.25, (**c**) PH0.5, (**d**) PH1, (**e**) PH2, (**f**) PH3, (**g**) PH5, and (**h**) PH7.

**Figure 4:**
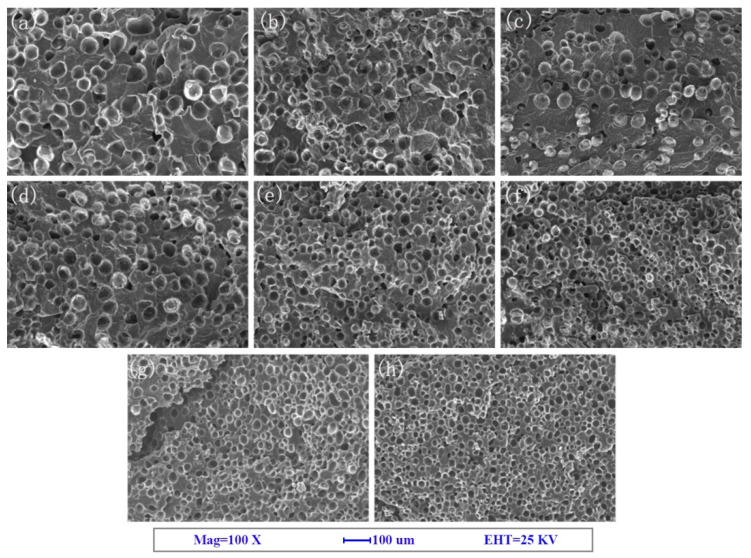
SEM images of the PP and PP + *β*-cyclodextrin (BC) (PB) composite foams: (**a**) PP, (**b**) PB0.25, (**c**) PB0.5, (**d**) PB1, (**e**) PB2, (**f**) PB3, (**g**) PB5, and (**h**) PB7.

**Figure 5:**
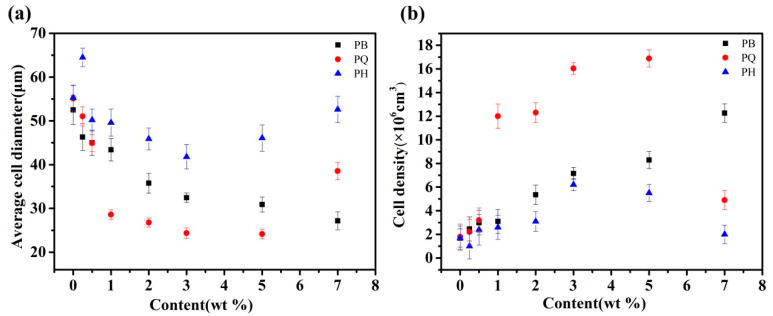
Average cell diameters (**a**) and cell densities (**b**) of the foamed PQ, PH, and PB.

SEM images of the fracture surface are shown in Figure 2, Figure 3 and Figure 4. The corresponding cell morphology parameters, including the average cell sizes and cell densities, are presented in Figure 5. As expected, the PP foam presented a poor cell structure, characterized by a large cell size and non-uniform cell distribution. The average cell size decreased with the addition of Q[6], HQ[6], and BC nanoparticles to the PP. Specifically, the cell size of the pure PP decreased from 55 μm to 26 μm when the PQ2 nanocomposite foam was used. The cell density increased from 1.9 × 106 cells/cm^3^ for the pure PP foam to 1.3 × 107 cells/cm^3^ for the composite foams. When the Q[6] content increased to 1.0 wt %, an obvious improvement in the cell structure was observed. However, further increments in Q[6] content did little to change the cell morphology.

When HQ[6] was added to PH at 3.0 wt %, the morphology of the cells improved. The cell diameter was 41.3 μm, and the cell density was 6.2 × 106/cm3. However, when the added HQ[6] exceeded 3.0 wt %, the PH cells began to deteriorate, and the cell diameter increased sharply. For example, when the content was 7.0 wt %, the cell parameter was close to that of the pure PP.

With the increase in BC content, the average size of the PB bubbles decreased and the cell density increased. When the maximum amount of BC was 7.0 wt %, the minimum average cell size of the PB was 27.1 μm and the maximum cell density was 1.22 × 10^7^/cm^3^.

### 3.2. Discussion on Nucleating Efficiency and Structure-Property Relationship

**Figure 6:**
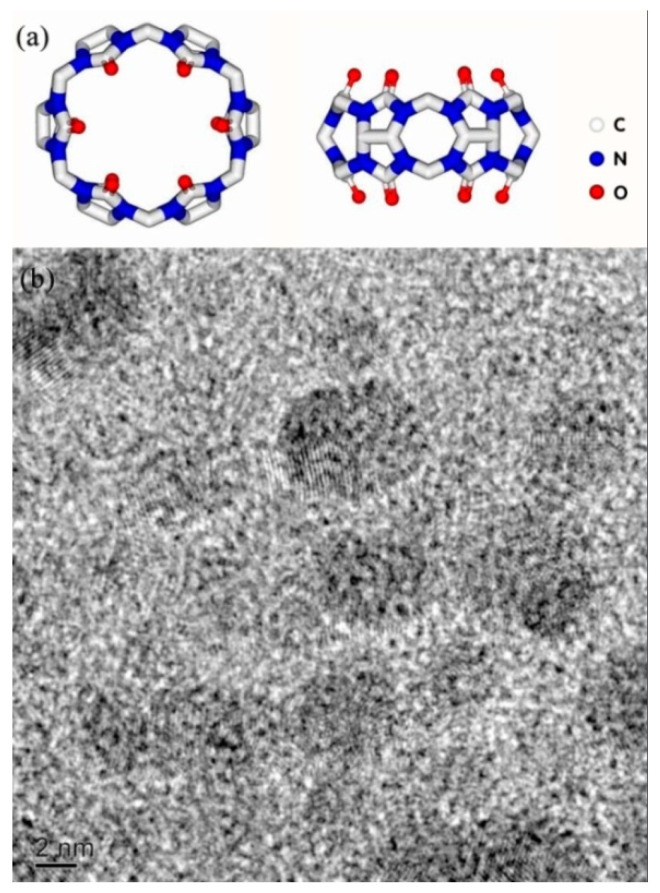
(**a**) Molecular structure and (**b**) TEM image of Q[6].

**Figure 7:**
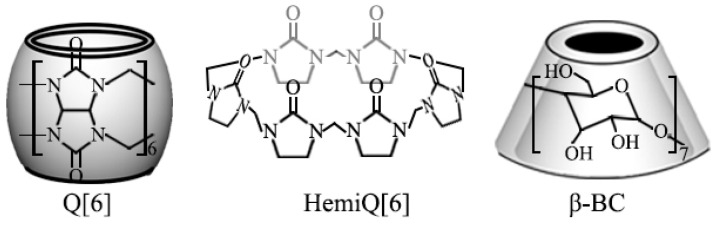
Molecular structures of Q[6], HQ[6], and BC.

Q[6] (Figure 6) is a macrocyclic compound synthesized with glycoluril and formaldehyde. It has a highly polarizable carbonyl-rich portal and a hydrophobic interior cavity. The structure of HQ[6] is similar to the structure of Q[6], i.e., it cuts halfway along the equator of the cage structure of Q[6]. The glycoside urea unit bridged by the methylene unit makes HQ[6] lose its structural rigidity and become a flexible macro-ring structure. Under the strong external force of melt shear, the material easily twists and deforms into a continuous phase interface with a radian. BC [25,26] is a cyclic oligosaccharide, consisting of glucopyranose units in a torus-like structure, with hydrophilism on the outer surface and relative hydrophobicity in their internal cavity. The molecular structures of the three cage compounds and TEM diagram of Q[6] are shown in Figure 7.

According to classical nucleation theory, the Gibbs free energy (Gibbs) barrier of gas conuclei can be expressed as

(2) $$\Delta G_{hom}=-\left(P_{bub}-P_{sys}\right)V_{g}+\sigma_{lg}A_{lg}$$

The Gibbs free energy barrier of heterogeneous nucleation can be expressed as follows:

(3) $$\Delta G_{het}=-\left(P_{bub}-P_{sys}\right)V_{bub}+\sigma_{\mathrm{lg}}A_{\mathrm{lg}}+\sigma_{sg}A_{sg}-\sigma_{sl}A_{sl}$$

where *P_bub_* is the gas pressure in the bubble; *P_sys_* is the ambient system pressure; *A* is the surface area; *σ* is the interface energy; and *l*, *g*, and *s* are the melt, gas, and solid phases, respectively.

When a gas is nucleated on a solid nucleating agent, the term *σ_sl_A_sl_* can reduce the energy barrier of the system, because no new generation is required at the solid–melt interface. When the boundary area, *A_sl_*, is fixed, the larger the interface energies of *σ_lg_* and *σ_sg_*, the larger the heterogeneous nucleation barrier, Δ*G_het_*. A traditional nucleating agent relies on *σ_sl_A_sl_* to reduce the nuclear barrier. The gas and melt interface energy, *σ_lg_A_lg_*, is the key factor that restricts the nuclear barrier. If *σ_lg_A_lg_* can be reduced, then the nucleation efficiency will improve.

Among the three cage compounds, Q[6] exerted a greater improvement effect on the PP at a low amount of addition, which means that Q[6] had the highest nucleation efficiency. On the one hand, BC contains many hydroxyl functional groups with a relatively strong molecular polarity. The polarity difference between the PP, which is a non-polar polymer, and BC was the largest in this study. Under the same experimental conditions, the interface energy value of BC as the nucleating agent system was the largest. Q[6] and HQ[6] are similar in chemical composition and have the same polarity. Their molecular polarity is weaker than that of BC. On the other hand, the hydroxyl groups in BC generate hydrogen bonds with one another, resulting in the “sealing end” of BC molecules and forming a special 3D structure. In this structure, one end of BC is open and the other end is nearly closed. Compared with Q[6], the BC with an approximate cone structure had a larger *A_lg_* value, i.e., Δ*G_het_*(BC) > Δ*G_het_*(Q[6]). Under the strong external force of melt shear, HQ[6] was easily twisted and deformed into a continuous phase interface, and Δ*G_het_*(HQ[6]) > Δ*G_het_*(Q[6]).

**Figure 8:**
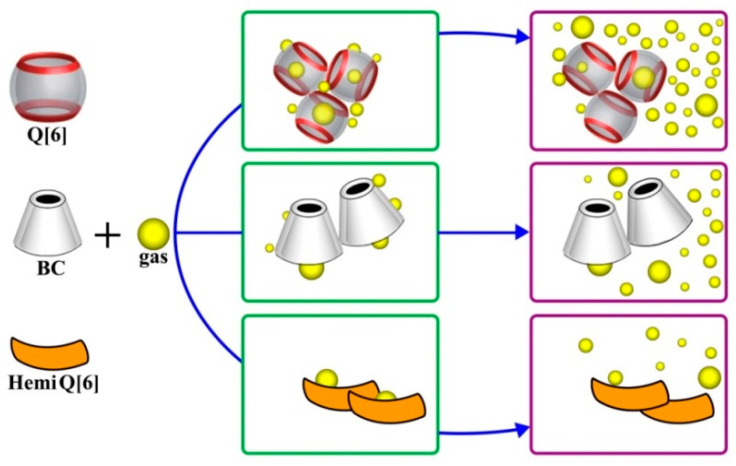
Schematic diagram of heterogeneous nucleation of Q[6], HQ[6], and BC.

In summary, the structural composition of Q[6] endows the material with a low surface density and reduces the gas nuclear energy barrier. However, HQ[6] and BC have high nuclear barriers and a relatively poor nucleation performance because of their high surface density and polarity (Figure 8).

**Figure 9:**
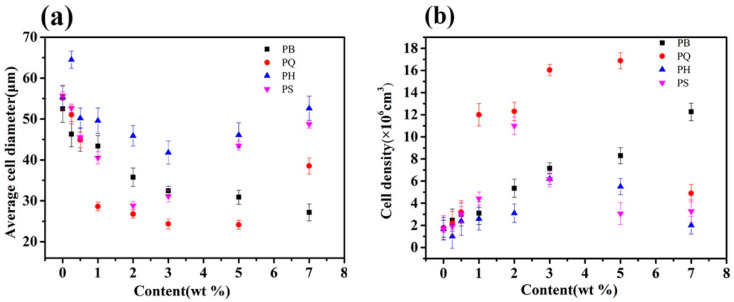
The average cell diameters (**a**) and cell densities (**b**) of the foamed PP composites with different contents.

**Figure 10:**
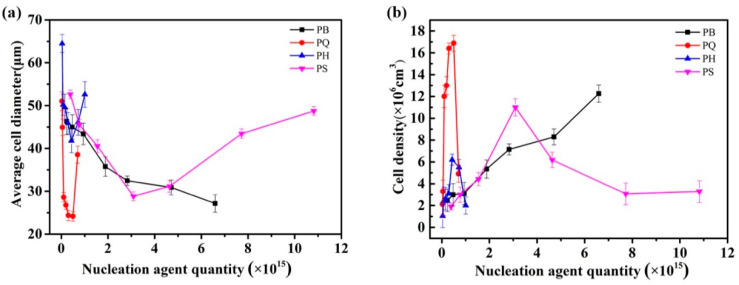
Average cell diameters (**a**) and cell densities (**b**) of the foamed PP composites with different nucleation agent quantities.

Silicon dioxide (SiO_2_), a traditional nucleation agent, was used to compare the nucleation performance of the organic caged and traditional nucleation agents (Figure 9 and Figure 10). SiO_2_ is an inorganic nucleating agent with a solid spherical structure, and it is commonly used to prepare polymer foaming materials. The composite of SiO_2_ and PP was labeled PS in this study. The weight of the nucleating agent added with PP in the experiment was converted into the number of particles for the scientific characterization of the nucleation performance of the various nucleating agents. Trends of the changes in average cell diameter and density with the number of nucleating agent particles were obtained and are shown in Figure 10.

The average cell diameter of PB decreased gradually with the increase in the number of nucleation sites. PQ, PH, and PS presented the same variation trends with the number of nucleation sites. Specifically, with the increase in the number of nucleation sites, the average cell diameter decreased and then increased, whereas the average cell density increased and then decreased. This result was obtained because the addition of the nucleating agent promoted the heterogeneous nucleation of cells. When the added content was increased, the agglomeration effect of the nucleating agent particles reduced the number of effective nucleation sites in the PP, thus diminishing cell improvement.

The improvement effect of HQ[6] on the PP cell morphology was limited. Compared with HQ[6], the PQ, PB, and PS series achieved a better improvement in PP cell morphology and obtained a smaller cell size and a higher cell density. Moreover, the number of nucleating particles required to achieve the same improvement differed. For example, when the PP cell size was reduced by 50%, the number of particles required for each nucleating agent differed by several times. The number of nucleation sites for PQ, PB, and PS was 0.19 × 1015, 6.5 × 1015, and 3.0 × 1015, respectively. Among the three series, PQ required the least nucleating particles to obtain the same bubble density, whereas PS and PB required the most. This experimental result proves that the nucleation efficiency of Q[6] was higher than that of the traditional nucleating agent, SiO2. According to the preceding theoretical analysis, SiO2 is a solid sphere, and its contact surface with the gas and melt is a continuous interface with a high density. It relies on the term *σslAsl* to reduce the nuclear barrier. However, the gas–melt interface energy term is the key factor that restricts the nuclear barrier of the bubble shape. Unlike in Q[6], which is composed of a single atomic layer, *Alg*(SiO2) > *Alg*(Q[6]). Therefore, the nucleation barrier Δ*Ghet*(SiO2) >Δ*Ghet*(Q[6] is required for gas nucleation on the surface. A many of nucleating agent particles are needed for SiO_2_ to achieve the same improvement effect.

### 3.3. In Situ Foaming Visualisation

The visual injection molding equipment developed by our research group can perform real-time monitoring of the growth process of cells in actual injection molding. The formation and growth of cells in the actual dynamic injection molding process of polymer foam materials can be determined by directly observing and comparing the results of actual injection molding.

In the current experiment, PP and the nucleating agent composites containing the same amount of nucleating agent particles were prepared using a mixer. The specific added content is shown in Table 1 (the particles are treated approximately as spheres).

**Figure 11:**
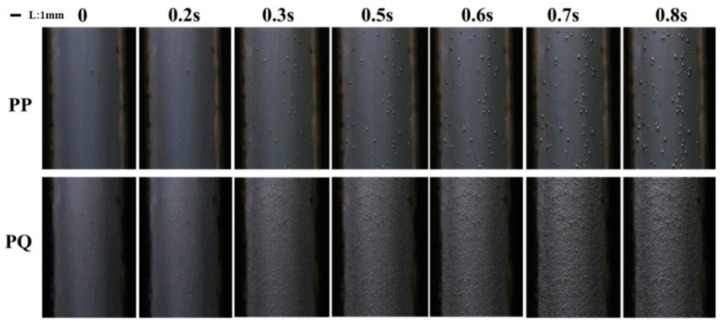
Pictures of the cells of the pure PP and the PQ composites changing over time.

Figure 11 shows images of the bubble growth of the pure PP and the PQ composites in the visual injection molding equipment. The growth of bubbles was relatively slow, and the number of cells was small in the injection molding process of pure PP. After the addition of nucleating agent Q[6], the bubble growth rate of PQ significantly increased. The number of PQ cells increased by nearly two orders of magnitude compared with the number of pure PP cells. This result indicates that the addition of a heterogeneous nucleating agent significantly reduced the nuclear energy barrier of gas, made the growth of cell nucleation easy, and increased the cell density significantly.

**Figure 12:**
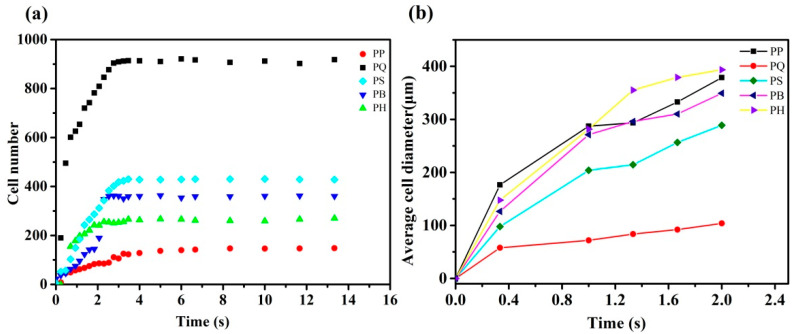
The average cell diameter (**a**) and cell density (**b**) of the foamed PP composites with different times.

Figure 12 shows the variation trends in the number and diameter of cells of the PP and PP composite systems over time. The number of cells in all of the experimental systems increased initially and then stabilized, which was a manifestation of gas nucleation from continuous growth to complete nucleation. The growth rate of the number of cells reflected the rate of nucleation and was related to the nuclear barrier of the system. The faster the growth, the easier the nucleation and the lower the nuclear energy barrier of the system. Hence, the slope of the bubble growth phase could be compared to that of the nuclear barrier. The higher the slope, the faster the nucleation and the lower the nuclear barrier. The slope of the bubble growth stage of the pure PP was the lowest, and the degree of bubble equilibrium was significantly lower than that of the other experimental systems, indicating that the nuclear energy barrier of the pure PP was the largest. After the addition of the same amount of nucleating agent particles, the growth rate of the number of balanced bubbles and the number of bubbles in each composite system became significantly higher than that of the PP. The PQ system had the highest slope and the fastest nucleation during the growth stage of the number of cells, followed by the PS, PB, and PH systems. The experimental results showed that Q[6] had the lowest nuclear energy barrier among all of the nucleating agents. A comparison of the number of equilibrium cells in each composite system showed that PQ had the largest number of cells, which was about nine times that of the pure PP. The number of cells increased and decreased successively in the order of PS, PB, and PH.

**Figure 13:**
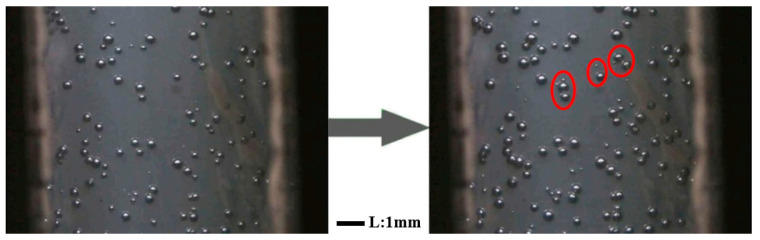
Photos of the secondary bubble growth of the PQ composites.

As shown in Figure 13, new cells were generated near the previously nucleated and growing cells, despite the rapid gas consumption from cell growth in these regions. This phenomenon was less pronounced when the pure PP sample was foamed. The cavity of Q[6] can also become a natural “air pocket”. As nucleated bubbles expand, their growth causes tangential stretching on the surface [27], resulting in a reduction in nearby pressure. *R*_cr_ and Δ*G*_het_ are reduced [28,29] accordingly to promote the nucleation of new bubbles and the growth of the original gas cavity, thereby increasing the final material bubble density. The nucleation efficiency of PQ with cavitation was significantly higher than that of the PP. The results of the experiment once again confirmed the conclusion that the cavity not only reduced the nuclear barrier, but also stimulated the growth of secondary bubbles. The in situ observation of the growth of the secondary bubbles revealed that the potential mechanism of nucleation was enhanced by the nucleating agents, and comprehensively explained the mechanism of heterogeneous nucleation promoted by fillers.

**Figure 14:**
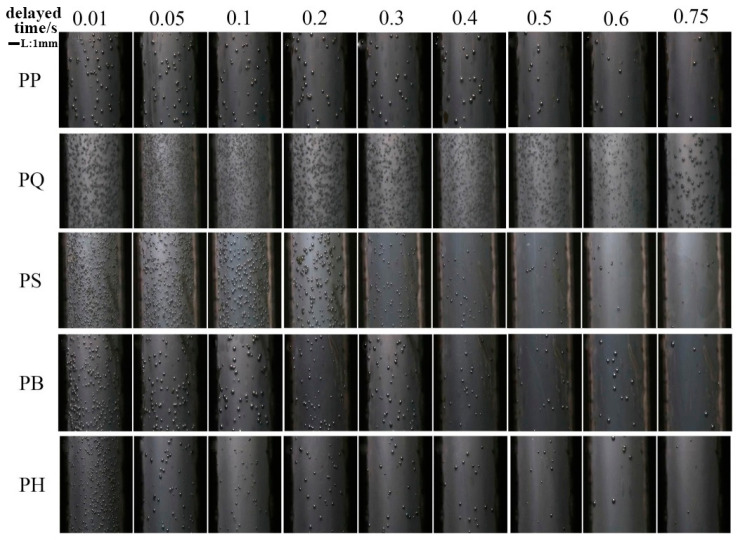
Pictures of the bubbles of the PP composite with different mold opening times.

**Figure 15:**
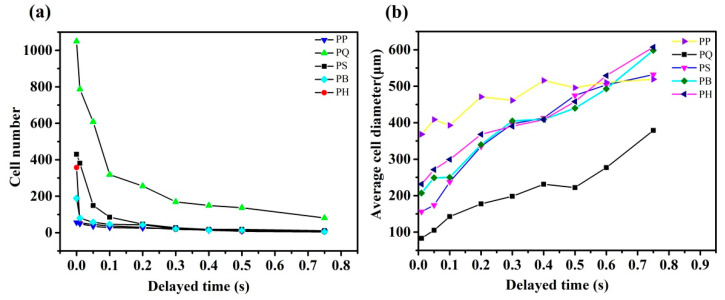
The cell number (**a**) and cell density (**b**) of the foamed PP composite with different mold opening times.

In addition, the visualized foaming behavior was observed under the same injection molding conditions, but with an extended mold opening time to verify the experimental conclusions further. The relation curve between the change in cell number and the delay time of each composite system is shown in Figure 14. At the end of the injection, when the mold opening time was prolonged, the temperature in the mold cavity decreased, and the melt viscosity increased. In other words, prolongation of the mold opening time increased the nuclear energy barrier and made bubble nucleation difficult. The higher the bubble reduction rate, the more difficult bubble nucleation became and the higher the nuclear energy barrier. As indicated in Figure 14 and Figure 15, the PQ system had the lowest rate of bubble number reduction, followed by the PS, PB, and PH systems. The pure PP system had the highest rate of bubble number reduction, which also reflected the order of the heterogeneous nuclear energy barrier of the systems. The nucleating agent of the PQ system had the lowest nuclear energy barrier among all of the nucleating agents, and this finding is consistent with the results of the microporous injection foam.

## 4. Conclusions

Analysis of the heterogeneous nucleation mechanism showed that *σ_lg_A_lg_* was the key factor that restricted bubble nucleation. When the value of *σ_lg_A_lg_* was reduced, the efficiency of nucleation improved. Organic cage compounds Q[6], HQ[6], and BC with different cavity structures were synthesized. A natural “cavity” was observed. This cavity was a large area of the “natural” gas/solid interface of Q[6], and it effectively reduced the value of *σ_lg_A_lg_*, decreased the heterogeneous nuclear barrier, and had the highest nucleation efficiency. The growth rule of the bubble obtained by visual injection molding was consistent with the conclusion from the actual injection molding. The conclusion that the low surface density of the nucleating agent could reduce the nuclear energy barrier and improve nucleation efficiency was verified again. This work provides a reference for the application of organic cage compounds in polymer foaming materials.

## Figures and Tables

**Table 1:** Parameters of nucleating agents.

	BC	Q[6]	HQ[6]	SiO_2_
density (g/cm^3^)	1.37	1.44	1.23	1.12
particle size (nm)	114	204	326	104
single particle volume (×10^−15^ cm^3^)	0.775	4.443	5.774	0.589
single particle weight (×10^−15^ g)	1.062	6.220	6.929	0.647
add weight per 100 g PP (g)	0.085	0.5	0.55	0.05
